# Genotyping of* IL-4* −590 (C>T) Gene in Iraqi Asthma Patients

**DOI:** 10.1155/2017/5806236

**Published:** 2017-03-12

**Authors:** Ihsan A. Hussein, Saddam H. Jaber

**Affiliations:** Department of Biology, College of Education for Pure Science-Ibn Al-Haytham, University of Baghdad, Baghdad, Iraq

## Abstract

This study is the first investigation in Iraq dealing with genotyping of* IL-4* −590 (C>T) gene, especially in Iraqi patients with asthma. We studied forty-eight blood samples collected from patients with asthma and compared with age-matched 25 healthy individuals as controls. The polymorphism results of* IL-4* −590 (C>T) gene by using amplification refractory mutation system (ARMS-PCR) showed the presence of* C* and* T* alleles and three genotypes (CC, CT, and TT). Interestingly the frequency of* C* allele and CC genotype was higher in patients with asthma in comparison with the same allele and genotype in control (*P *1 × 10^−6^). This increase was associated with an increased risk factor of asthma (odds ratio [OR] 9.21; 95% confidence interval [CI] 3.58–23.71). Genotypes analysis by using Hardy-Weinberg distribution showed no significant differences between patients with asthma and healthy subjects. In conclusion, the increasing risk of asthma was associated with* C* allele and the CC genotype and these are revealed as etiological fraction with risk by having this disease, while the* T* allele percentage ratio in controls was higher when it is compared with asthma patients suggesting that these alleles have a protective effect (preventive fraction).

## 1. Introduction

Asthma is a complex chronic inflammatory disease that results from the interaction between genetic predisposition and environmental factors [1]. More than 300 million persons worldwide were affected by this airways disease, with approximately 250,000 annual deaths as a result [2]. It has also been estimated that the number of asthmatic patients will increase by 2015 for more than 100 million [3]. Multiple interacting genes have been involved in causing this disease and contributing to the disease pathogenesis and other genes have a protective effect. Each gene may have its own tendency to be influenced by environment factors [4]. However, the identified asthma susceptibility genes have increased rapidly over the last 5 years, especially when genome wide association (GWA) study approach has been applied [1]. Interleukin 4 was originally discovered as a low molecular weight T cell-derived polypeptide of 129 amino acids, which is encoded by the* IL-4* gene located on chromosome 5q23.31:* IL-4* [5]. Closer to this chromosomal region (5q31-q33), asthma and atopy susceptibility gene(s) have been mapped by numerous genetic studies in several ethnic populations: Dutch [6, 7], Amish [8], American Caucasian [9], Hutterite [10], and British [11, 12]. The role that interleukin-4 plays in the pathogenesis of asthma has been indicated from actively sensitized IL-4 knockout mice [13, 14]. Studies on T helper 2 (Th2) cytokines in asthma have focused on IL-4 and IL-5 and this is due to the crucial role of these two cytokines in Th2 generation responses in a variety of animal models. IL-4 is essential for the maturation of native T cells toward Th2 cells and production of IgE [15, 16]. Polymorphisms in genes play key roles in regulation of cytokines expression; for example, substitution of C by T at the position −590 of* IL4* gene has been reported to be associated with a reduced* IL4* expression, while the TT genotype upregulated the production of this cytokine [17]. This work is the first investigation in Iraq that dealt with genotyping of* IL4* −590 (C>T) in a sample of Iraqi asthmatic patients with the aim to determine its susceptibility role.

## 2. Material and Methods

### 2.1. Subjects

Forty-eight asthma patients and 25 controls (healthy) subjects were enrolled in the study. The patients were referred to the Al Zahra Center for Allergy and Asthma in Baghdad, Iraq, for diagnosis and treatment. The diagnosis was made by the consultant medical staff according to international criteria. All cases and controls were at the age range 10–65 years. Anticoagulants EDTA tubes were used for collecting the peripheral blood for DNA isolation.

### 2.2. Genotyping

DNA from venous blood was isolated using ReliaPrep™ Blood gDNA Miniprep System kit (Promega). The* IL-4* gene at position −590 (C>T) was genotyped using the amplification refractory mutation system (ARMS-PCR) approach. Primers for this position were used according to [18] and synthesized at Alpha DNA Company (Canada) (Table 1).

### 2.3. ARMS-PCR

ARMS-PCR approach was used for genotyping of* IL4* −590 (C>T). AccuPower® PCR PreMix (Bioneer, Korea) was prepared according to [18] with some modifications. The primers and DNA template were added to PCR PreMix tubes and the final volume for PCR reaction was made up to 20 *μ*L with nuclease-free water (Table 2). The reaction mixers were placed in thermal cycler (Esco, Singapore), and PCR conditions for the all reaction mixers are described in Table 3.

The products of PCR were resolved on 1.5% agarose gels. The DNA 100 bp ladder (Bioneer, Korea) was also loaded on the agarose gel. Two microliters of bromophenol blue dye was loaded with all reaction mixer samples. The gel electrophoresis was performed by using 75 V for 2 hrs and stained with ethidium bromide (Promega, USA) for 30 min. The gel was documented with gel documentation system (Biocom, USA).

### 2.4. Statistical Analysis

Percentage frequencies and significant differences data between patients and controls were given by Fisher's exact test. The odds ratio (OR) and confidence intervals (CI) were analyzed by using Compare 2 Ver.3.04 program designed by J. H. Abramson/2003–2013. Deviations from Hardy-Weinberg were tested using an exact test available at http://www.had2know.com/academics.html.

## 3. Results and Discussion 

**Figure 1:**
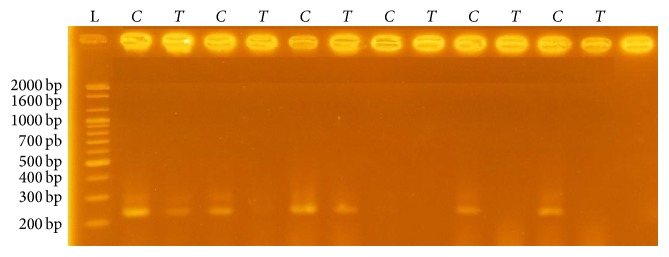
Gel electrophoresis for the* IL-4* gene −590 (C>T) showing the* C *and* T* alleles in some asthma patients. (Gel electrophoresis was done by using 1.5% agarose gel concentration, 75 volts for 2 hours. Presence of one band in* C *lane and absence of this band in* T* lane refer to the genotype CC. In contrast, presence of one band in* T *lane and absence of this band in* C* lane refer to the genotype TT. Presence of two bands in both lanes refers to the genotype CT.)

By using ARMS-PCR, the polymorphism of* IL4* −590 (C>T) showed the presence of* C *and* T* alleles and three genotypes (CC, CT, and TT) by using the specific* C*, specific* T, *and reverse primers (Figure 1).

Genotype frequencies of* IL4*-590 (C>T) were in a good agreement with Hardy-Weinberg equilibrium in asthma patients and controls, as there were no significant differences between observed and expected genotype frequencies (Table 4).

The* C *allele showed a significant increased frequency in patients compared to controls (92.71 versus 58%). In contrast, the* T* allele frequency was significantly higher in controls (42%) than in patients (7.29%). The OR for* C* and* T* alleles were 9.21 and 0.11, respectively. Such results suggest that* C* allele has a predisposing effect, while* T* allele has a protective effect (Table 5). In terms of genotype frequencies, CC genotype was observed to have a significant increased frequency in patients compared to controls (87.5 versus 32%), while CT and TT genotype frequencies were significantly decreased in patients (10.42% and 2.08%, respectively) compared to controls (52% and 16%, respectively). The OR for CC, CT, and TT genotypes was 14.88, 0.11, and 0.11, respectively (Table 6). An increased risk of asthma might be associated with CC genotype, while the genotypes CT and TT are thought to have a protective effect from asthma.

Studies of candidate-genes have examined the involvement of many genes in asthma and allergy and demonstrated a role for more than 100 loci. Several themes that regard biology and pathogenesis of these diseases have elucidated by these studies. Traditional linkage analyses revealed a small number of genes that are associated with asthma or allergy [19].

Cytokines are key mediators that regulate innate and adaptive immune responses, which are also subjected to the effect of factors such as infection, inflammation, and hormonal condition. However, cytokine gene polymorphisms have a role in the expression of cytokines by immune cells [20]. The* C* allele frequency in asthma patients was higher in comparison with* T* allele and this refers to the role of CC genotype in the development of the disease, but these results need more evidences for confirming and also need more immunological studies such as detecting the level of IL-4 in sera of asthma patients. In a Dutch study of gene-gene interaction in asthma, Howard et al. [21] observed significant associations of atopy and asthma with several* IL4RA *polymorphisms, including S478P and total serum IgE levels (*P* = 0.0007). A further study carried out by Xiaoyan et al. [22] investigated six gene loci (one of them is of* IL4*-590), and the authors suggested that these genes make little contribution to the development of asthma in children of Chinese Han nationality.

## 4. Conclusions

In conclusion, the increasing risk of asthma was associated with* C *allele and the CC genotype, while the* T* allele and CT and TT genotypes were present more in healthy subjects which are thought to have a protective effect (preventive fraction). These alleles dominating in special population compared to others at genetic levels could be used as an indicator of genetic biomarkers that effect on either pathogenesis or protection from the diseases.

## Figures and Tables

**Table 1:** Primers for *IL-4* −590 (C>T) genotyping.

Primer	Sequence (5′ → 3′)	Product size (bp)
T allele	5′-ACACTAAACTTGGGAGAACATTGTT-3′	216
C allele	5′-ACACTAAACTTGGGAGAACATTGTC-3′	248
Reverse	5′-GAATTTGTTAGTAATGCAGTCCTCC-3′	—

**Table 2:** PCR mix reaction for genotyping of *IL-4* gene position −590 (C>T).

Component	Volume (*μ*l)	Final concentration
Each primer (T or C allele + reverse)^*∗*^	2	1 *μ*M
DNA template	5	100 ng
Nuclease-free water	11	—
Final volume	20	—

^**∗**^T and reverse primers were used for detecting *T* allele; *C* and reverse primers were used for detecting *C* allele.

**Table 3:** PCR conditions for genotyping of *IL-4* gene position −590 (C>T).

Steps	Temperature (°C) and cycles		Time (sec)
Denature template	96		60
First initial denaturation	95	10 cycles	15
First annealing	65		50
First extension	72		40
Second initial denaturation	95	20 cycles	50
Second annealing	59		50
Second extension	72		50
Final extension	72		7 min
Incubation	4		5 min

**Table 4:** Hardy-Weinberg distribution of *IL4* −590 (C>T) genotypes in asthma patients and controls.

Gene	Genotype	Observed asthma patients number (%)	Expected asthma patients number (%)	Observed controls number (%)	Expected controls number (%)
*IL-4* −590 (C>T)	CC	42 (87.5)	(85.95) 41.26	8 (32)	(33.64) 8.41
	CT	5 (10.42)	(13.52) 6.49	13 (52)	(48.72) 12.18
	TT	1 (2.08)	(0.53) 0.26	4 (16)	(%17.64) 4.41
	*P* value	0.1118		0.7364	

**Table 5:** Allele frequencies of *IL4* −590 (C>T) in asthma patients and controls.

Gene position	Allele	Asthma patients number (%)	Controls number (%)	OR (95% CI)	*P* value
*IL-4* −590 (C>T)	*C*	(92.71) 89	(58) 29	9.21 (IC = 3.58−23.71)	1 × 10^−6^^*∗*^
	EF	0.826			
	*T*	(7.29) 7	(42) 21	0.11 (IC = 0.04−0.28)	1 × 10^−6^^*∗*^
	PF	0.374			

OR = odds ratio, CI = confidence intervals, EF = etiological fraction, and PF = preventive fraction. ^*∗*^Significant differences at *P* < 0.05 level by using Fisher's test.

**Table 6:** Frequency distribution of *IL4* −590 (C>T) genotypes in asthma patients and controls.

Gene	Genotype	Asthma patients number (%)	Controls number (%)	OR (95% CI)	*P* value
*IL-4* −590 (C>T)	CC	42 (87.5)	8 (32)	14.88 (IC = 4.57−48.45)	2.5 × 10^−6^
	EF	0.816			
	CT	5 (10.42)	13 (52)	0.11 (IC = 0.03−0.35)	3.1 × 10^−4^
	PF	0.464			
	TT	1 (2.08)	4 (16)	0.11 (IC = 0.01−1.03)	0.044
	PF	0.142			

OR = odds ratio, CI = confidence intervals, EF = etiological fraction, and PF = preventive fraction.
